# Monitoring autochthonous lung tumors induced by somatic CRISPR gene editing in mice using a secreted luciferase

**DOI:** 10.1186/s12943-022-01661-2

**Published:** 2022-10-03

**Authors:** Nastasja Merle, Sabrina Elmshäuser, Florian Strassheimer, Michael Wanzel, Alexander M. König, Julianne Funk, Michelle Neumann, Katharina Kochhan, Frederik Helmprobst, Axel Pagenstecher, Andrea Nist, Marco Mernberger, André Schneider, Thomas Braun, Tilman Borggrefe, Rajkumar Savai, Oleg Timofeev, Thorsten Stiewe

**Affiliations:** 1 Institute of Molecular Oncology, Universities of Giessen and Marburg Lung Center (UGMLC), Member of the German Center for Lung Research (DZL), Philipps-University, Marburg, Germany; 2 Clinic of Diagnostic and Interventional Radiology, Philipps-University, Core Facility 7T-small animal MRI, Marburg, Germany; 3 Mouse Pathology and Electron Microscopy Core Facility, Department of Neuropathology, Philipps-University, Marburg, Germany; 4 Genomics Core Facility, Philipps-University, Marburg, Germany; 5 Department of Cardiac Development and Remodeling, Member of the German Center for Lung Research (DZL), Max-Planck-Institute for Heart and Lung Research, Bad Nauheim, Germany; 6 Department of Biochemistry, Justus Liebig University, Giessen, Germany; 7 Max-Planck Institute for Heart and Lung Research, Member of the German Center for Lung Research (DZL), Member of the Cardio-Pulmonary Institute (CPI), Bad Nauheim, Germany; 8 Institute for Lung Health (ILH), Justus Liebig University, Giessen, Germany

**Keywords:** Autochthonous mouse tumor, Lung cancer, Orthotopic tumor, Luciferase, CRISPR, Adenovirus

## Abstract

**Background:**

In vivo gene editing of somatic cells with CRISPR nucleases has facilitated the generation of autochthonous mouse tumors, which are initiated by genetic alterations relevant to the human disease and progress along a natural timeline as in patients. However, the long and variable, orthotopic tumor growth in inner organs requires sophisticated, time-consuming and resource-intensive imaging for longitudinal disease monitoring and impedes the use of autochthonous tumor models for preclinical studies.

**Methods:**

To facilitate a more widespread use, we have generated a reporter mouse that expresses a Cre-inducible luciferase from *Gaussia princeps* (GLuc), which is secreted by cells in an energy-consuming process and can be measured quantitatively in the blood as a marker for the viable tumor load. In addition, we have developed a flexible, complementary toolkit to rapidly assemble recombinant adenoviruses (AVs) for delivering Cre recombinase together with CRISPR nucleases targeting cancer driver genes.

**Results:**

We demonstrate that intratracheal infection of GLuc reporter mice with CRISPR-AVs efficiently induces lung tumors driven by mutations in the targeted cancer genes and simultaneously activates the GLuc transgene, resulting in GLuc secretion into the blood by the growing tumor. GLuc blood levels are easily and robustly quantified in small-volume blood samples with inexpensive equipment, enable tumor detection already several months before the humane study endpoint and precisely mirror the kinetics of tumor development specified by the inducing gene combination.

**Conclusions:**

Our study establishes blood-based GLuc monitoring as an inexpensive, rapid, high-throughput and animal-friendly method to longitudinally monitor autochthonous tumor growth in preclinical studies.

**Supplementary Information:**

The online version contains supplementary material available at 10.1186/s12943-022-01661-2.

## Background

Cancer is caused by mutations in tumor suppressors and proto-oncogenes, most of which are acquired by somatic cells during life-time. The identity and combination of the affected genes defines genetic cancer subtypes with distinct morphology, aggressiveness, metastatic potential, clinical prognosis and, probably most importantly, with different therapeutic vulnerabilities. In lung cancer, for example, small cell lung cancer (SCLC) almost universally displays combinations of inactivating mutations in the tumor suppressor gene *TP53* and one or more of the *RB* family pocket proteins [1]. In contrast, non-small cell lung cancer (NSCLC) is characterized by activating mutations affecting proto-oncogenic receptor tyrosine kinases or downstream signal transducers, offering personalized therapy options with oncogene-targeted drugs such as tyrosine or MAP kinase inhibitors [2]. The value of animal models for preclinical therapy studies therefore critically depends on how accurately mouse tumors resemble the complex and diverse genetics of human tumors [3].

Among the broad spectrum of animal cancer models, xenograft models based on patient-derived primary tumor tissues or human cancer cell lines obviously model the genetics of human tumors most precisely and have become especially popular for therapy studies, as tumors often grow rapidly within a few weeks. When grafted on the flank of mice, tumor growth is directly visible and measurable with calipers which facilitates the establishment of large animal cohorts with tumors of similar stage. However, being transplanted and grown in an immunocompromised host, xenografts fail to develop the characteristic tumor microenvironment (TME) that is shaped by stage-specific, reciprocal interactions with stromal and immune cells [4–7]. As the TME promotes metastatic tumor progression, protects tumors from drugs and immune attack, and presents itself numerous therapeutic targets, immunodeficient xenograft models have limited value for the preclinical evaluation of many TME-targeted treatments including, for example, immune checkpoint inhibitors [5–7].

At the other end of the spectrum are non-transplanted, autochthonous tumors developing orthotopically from single normal cells that were transformed in situ by engineered mutations. In such genetically engineered mouse models (GEMM) tumorigenic mutations are most commonly introduced into the germline of mice, with various conditional approaches allowing for temporal and spatial expression control. In a laborious and time-consuming process, multiple different germline alleles are combined by cross-breeding to obtain experimental animals with the desired complex genotype. In the course, a majority of mice are sacrificed because of an unwanted genotype, which is constantly raising major ethical concerns. More recent advances with in situ mutagenesis of somatic cells using CRISPR technologies have allowed to directly introduce multiple defined tumorigenic mutations into living mice [8–10]. Circumventing germline modifications, somatic gene editing avoids the ethical problems of inefficient breeding schemes and, at the same time, models the natural course of tumorigenesis originating from a single, somatically mutated cell even better.

A technical hurdle of in vivo mutagenesis remains the efficient delivery of the gene editing machinery. Currently, gene transfer by viral vectors such as lentiviruses, adenoviruses (AV) or adeno-associated viruses is most efficient in many models, but limited by the large size of expression cassettes for CRISPR nucleases such as SpCas9, sgRNAs and accessory proteins like Cre, which together exceed the cargo packaging capacity of many common vectors [9]. This problem can be circumvented by split-approaches that separate the effectors into different vectors [11], by switching to naturally shorter or size-optimized CRISPR enzymes [12], by newer vector generations with higher cargo capacity [13] or by providing some of the CRISPR components, such as Cas9, via a conditional, Cre-inducible, germline transgene [14]. For example, adenovirus- or AAV-mediated transfer of CRISPR effectors targeting various tumor suppressor genes was successfully applied to engineer SCLC in mice, showing that co-mutations in the RB-family members *Rbl1* and *Rbl2* [15], the lysine demethylase *Kdm5a/Rbp2* [16] or Notch receptors *Notch1* and *Notch2* [17] enhance SCLC development initiated by combined *Trp53* and *Rb1* mutations.

Because genetically-altered cells progress in situ through different stages of tumorigenesis, involving the accumulation of secondary cooperating mutations and development of immune escape strategies, tumor growth is typically much slower, more variable in time and between animals, and consequently experimentally less predictable. Therapy studies using autochthonous tumors therefore require tools for longitudinal monitoring to identify mice with tumors of an appropriate stage and enroll them into the treatment protocol at the optimal time point.

Multiple methods for longitudinal tumor monitoring in animals have been developed, most of which rely on imaging technologies such as computed tomography, magnetic resonance imaging, positron-emission tomography, single-photon emission computed tomography, ultrasonography and optical imaging of bioluminescence or fluorescence [18]. Notably, these sophisticated technologies do not only require expensive equipment and a highly trained staff, imaging is also time-consuming and requires anesthesia for immobilization of the animal. In addition, contrast agents, radioactive or fluorescent tracers or luminescence substrates are often administered systemically to improve sensitivity or signal specificity. All these factors not only considerably increase the complexity of the experiment, but also contribute significantly to animal burden and therefore strongly limit the examination frequency.

An alternative tumor monitoring method uses secreted luciferases that are released by cells into the environment and which exhibit higher luminescence activity and stability than conventional luciferases [19]. Similar to clinical tumor markers, such as prostate-specific antigen, which are routinely used in patients for cancer screening or therapy monitoring, xenograft growth induced by transplanted tumor cells expressing a secreted luciferase from the marine copepod *Gaussia princeps* (GLuc) can be quantitatively monitored based on GLuc activity levels determined ex vivo in small-volume blood or urine samples [20–26]. Importantly, as GLuc secretion is an active energy-consuming process and the half-life of GLuc in circulation is only approximately 10 min [21], GLuc activity in the blood is specifically measuring the amount of viable tumor cells in the organism, thereby excluding both necrotic cells and stromal cells that contribute to the tumor volume measured by morphological imaging techniques [27]. While secreted luciferases have become increasingly powerful for monitoring transplanted tumors, it has so far not been possible to exploit their scientific and animal welfare advantages for monitoring the growth of non-transplanted, autochthonous tumors in germline or somatic GEMMs.

**Fig. 1:**
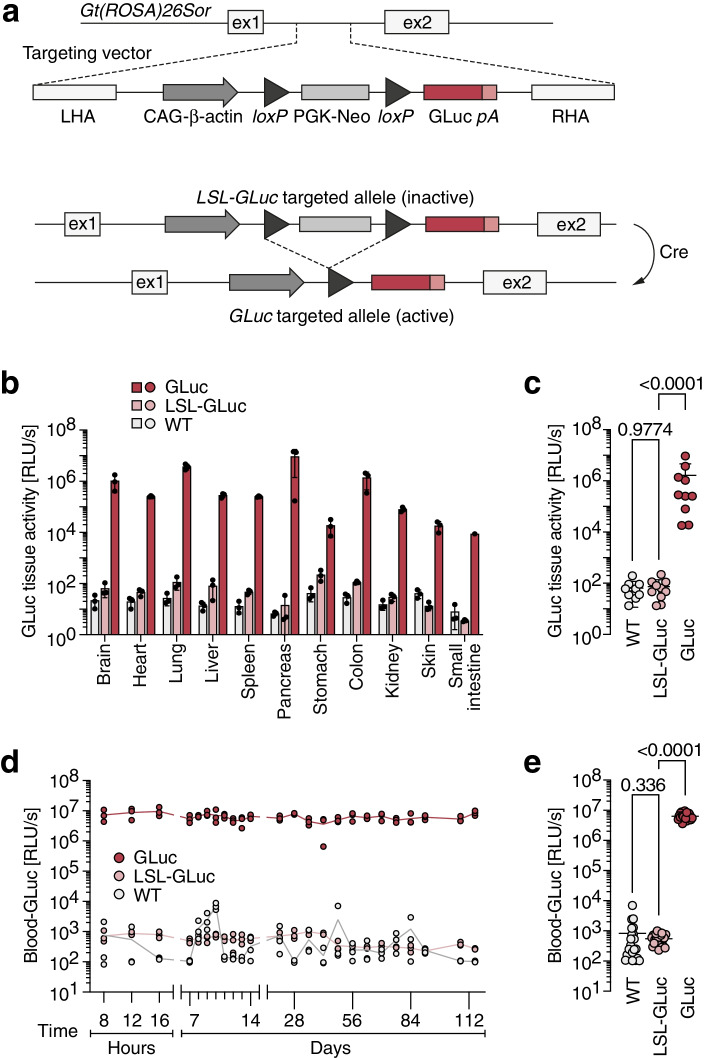
Conditional GLuc reporter mice. **a** Targeting strategy for *Gt(ROSA)26Sor*^*tm2(CAG−GLuc)*^ mice: insertion of a GLuc cDNA expression cassette controlled by the cytomegalovirus early enhancer/chicken beta actin (CAG) promoter and a *loxP*-flanked transcriptional stop cassette (LSL) into intron 1 of the *Gt(ROSA)26Sor* gene locus. **b** GLuc activity measured in organ lysates of mice with indicated genotypes (*n* = 3 biological replicates). **c** GLuc tissue activity. Each data point represents one tissue type (*n* = 11 tissues; P-values from Dunnett’s multiple comparisons test). **d, e** Level and temporal stability of GLuc activity in blood plasma of mice with indicated genotypes. **d** Time course, *n* = 3 mice per genotype. **e** Time average ± SD with data points representing individual time points (*n* = 24 time points; *P*-values from Dunnett’s multiple comparisons test

Here we describe a flexible and easy-to-use toolkit that combines the induction of genetically-defined autochthonous tumors by adenoviral CRISPR vectors with GLuc as a blood-based tumor marker for longitudinal disease monitoring. For this, we have first generated a reporter knock-in mouse *Gt(ROSA)26Sor*^*tm2(CAG−GLuc)Thst*^ containing a Cre-inducible GLuc transgene (Fig. 1a). Second, we have established a cloning system for rapid and flexible assembly of adenoviral CRISPR vectors (CRISPR-AVs) expressing both CRISPR effectors (SpCas9 and gene-specific sgRNAs) and Cre recombinase in an adenoviral vector backbone. Using lung cancer as a model, we demonstrate that infection of mouse lungs with such CRISPR-AVs induces tumor-initiating cancer gene mutations and simultaneously labels the incipient cancer cells with GLuc, so that cancer growth can be monitored over time by measurement of GLuc activity as a tumor marker in small-volume blood samples.

## Methods

### Animal experiments

All mouse experiments were performed according to the German Animal Welfare Law (TierSchG) and were approved by the local authorities (Regierungspräsidium Gießen and Darmstadt). Mice were housed in specific-pathogen free conditions, on a 12-h light/dark cycle and fed with standard housing diet (Altromin) receiving water ad libitum.

*Generation of* STOCK-*Gt(ROSA)26Sor*^*tm2(CAG−GLuc)Thst*^ (LSL-GLuc) *reporter mice.* Transgenic mice conditionally overexpressing GLuc luciferase in tumor cells are based on a modified ROSA26 targeting approach replacing the splice acceptor site of pBigT by a CAG promoter [28]. GLuc open reading frame was PCR amplified to introduce NheI and NotI sites, and cloned into the corresponding sites of the pBigT-CAG vector. Thereafter, the generated pBigT-CAG-GLuc cassette was cloned into PacI and AscI sites of the pRosa26-PA construct. The final construct was linearized by KpnI and electroporated into V6.5 F1 hybrid ES cells. Targeted stem cell clones were selected by G418 treatment and subsequently screened by Southern blotting using a 5’ external probe combined with EcoRV digestion. Positive ES cell clones were injected into C57BL/6 blastocysts. The resultant chimeras were backcrossed to C57BL/6 mice and maintained on a mixed C57BL/6/129 background.

Other mouse strains used are: 129S2*SvHsdThst*, STOCK-*Gt(ROSA)26Sor*^tm1(CAG−cas9*,−EGFP)Fezh^*/JThst* (LSL-Cas9) [14]*,* B6.129-*Gt(ROSA)26Sor*^tm2(ACTB−Luc)Tyj^/NciThst (LSL-FLuc) [29], B6.129S/Sv-*Kras*^tm4Tyj^/JThst (LSL-KrasG12D) [30], B6.Cg-Tg(IghMyc)22Bri/JThst (EµMyc) [31], C.129S4-*Rag2*^*tm1.1Flv*^* Il2rg*^*tm1.1Flv*^/JThst (Rag2γ) [32]. For removal of the LSL-cassette and ubiquitous luciferase expression, LSL-GLuc and LSL-FLuc mice were crossed with 129-Tg(Prm-cre)58Og/JThst (Prm-Cre) mice [33] yielding *Gt(ROSA)26Sor*^+/GLuc^ (GLuc) and *Gt(ROSA)26Sor*^+/FLuc^ (FLuc) mice, respectively.

*Transplanted lymphoma model.* For in vivo labelling of Burkitt-like lymphoma cells with luciferases, EµMyc transgenic males were crossed with heterozygous GLuc females to obtain double transgenic EµMyc;GLuc males that were bred with homozygous FLuc females. Compound transgenic EµMyc;GLuc/FLuc mice were monitored for lymphoma development. Lymphoma cells were isolated from lymph nodes and spleens of terminally sick mice and 10^6^ living cells were transplanted via tail vein injection into immunodeficient Rag2γ recipient mice. For chemotherapy of lymphoma, mice received a single i.p. injection of 300 mg/kg cyclophosphamide.

*Autochthonous lung cancer models.* For induction of lung cancer, mice were intratracheally injected with adenoviral vectors as described previously [34]. Briefly, mice were anesthetized by intraperitoneal injection of ~ 25 mg/kg ketamine and ~ 0.6 mg/kg medetomidine and maintained at 37 °C during anesthesia. Mice were intubated with a 20G catheter (B.Braun) and 1 × 10^9^ PFU/mouse of purified AV (ViraQuest Inc.) was applied in a volume of 50 µl. AVs were diluted in Minimal Essential Medium (MEM, Sigma) and 2 M CaCl_2_ was added to a final concentration of 10 mM. After complete inhalation, the catheter was removed, mice were kept warm and monitored for breathing and recovery. Anesthesia was partially antagonized using ~ 1.5 mg/kg atipamezole and mice were transferred to individually ventilated cages (IVC). For induction of lung adenocarcinoma, a *Kras*^+/LSL−G12D^;*Rosa26*^LSL−GLuc/LSL−FLuc^ mouse and a *Rosa26*^LSL−GLuc/LSL−FLuc^ control were infected with AV-Cre (ViraQuest Inc.). For induction of SCLC, wild-type, *Rosa26*^LSL−GLuc^ or *Rosa26*^LSL−Cas9/LSL−GLuc^ mice were infected with CRISPR-AVs.

Mice were analyzed by MRI and BLI using an IVIS 100 Imaging System (Xenogen), an In Vivo Xtreme II System (Bruker) or 7 T Clinscan 70 /30 USR (Bruker) as previously described [34, 35]. Mice were anesthetized with isoflurane. Bioluminescence was recorded 5 min after intraperitoneal injection of 200 µl D-luciferin (15 mg/ml in PBS, BioVision).

### Cell culture

The murine NIH 3T3 cell line was obtained from the American Tissue Collection Center (ATCC), Ad293 cells from Agilent. Primary dermal fibroblasts were isolated from LSL-Cas9 and LSL-GLuc mice: ear biopsies were minced and digested with 1000 U/ml collagenase Ia (Sigma) overnight at 4 °C followed by a 45 min treatment with 0.025% Trypsin solution (Sigma) at 37 °C. Disaggregated tissue was resuspended in 5 ml Dulbecco’s Modified Eagle Medium (DMEM, Thermo Fisher) supplemented with 20% fetal bovine serum (FBS, Sigma-Aldrich) and cultured until immortalization. For further experiments, cell lines were cultivated in a humidified atmosphere at 37 °C and 5% CO_2_ using DMEM supplemented with 10% FBS, 100 U/ml penicillin and 100 µg/ml streptomycin (Life Technologies).

*Generation of a NIH3T3-Cas9 cell line.* NIH3T3 cells were infected with the lentivirus lentiCas9-Blast (Addgene #52962) and selected with 20 µg/ml Blasticidin (Invivogen) starting 2 days post infection for 3 days until resistant cell clones were established. Infectious lentiviral particles were produced as previously described [36].

*Generation of murine SCLC cell lines.* Tumors were excised, washed twice in ice-cold PBS (Thermo Fisher), minced and digested in 2 ml of 0,025% Trypsin/EDTA (Sigma) at 37 °C for 30 min, resuspended in 5 ml of Roswell Park Memorial Institute 1640 Medium (RPMI 1640, Gibco) supplemented with 10% FBS and 100 U/ml penicillin and 100 µg/ml streptomycin. Cells were observed daily and medium was subsequently replaced every 3 days until a stable cell line was established.

### Adenoviral CRISPR vectors

Single-guide RNAs (sgRNAs) targeting genes of interest were designed and cloned into the pSpCas9(BB)-2A-Puro vector (Addgene #62988) using Golden Gate Cloning as described [24]. The following sgRNAs were used: *Trp53*: 5’-CAT AAG GTA CCA CCA CGC TG-3’, *Rb1*: 5’-GAA CAG ATT TGT CCT TCC CG-3 ‘, *Rbl2*: 5’-CCC GTG AGT CGA GTT GGT GT-3 ‘, Control: 5’-GGG CGA GGA GCT GTT CAC CG-3’. The pSpCas9(BB)-2A-Puro gRNA containing plasmids were used as template for PCR amplification of the U6-sgRNA region using Q5® High Fidelity DNA Polymerase (NEB). For directional Golden Gate Assembly of multiple sgRNA amplicons, BbsI restriction sites and unique 4 bp overhangs were added to the primers, the following primers were used: sgRNA1 forward 5’- GGT GAA GGA AGA CTC GGC TGA GGG CCT ATT TCC CAT G-3’; sgRNA1 reverse 5’- GGT GAA GGA AGA CTC CAA AAA AGC ACC GAC TCG G-3’, sgRNA2 forward 5’-GGT GAA GGA AGA CGT TTT GAG GGC CTA TTT CCC ATG-3’; sgRNA2 reverse 5’- GGT GAA GGA AGA CGT CCC TCA AAA AAG CAC CGA CTC GG-3’, sgRNA3 forward 5’-GGT GAA GGA AGA CTG AGG GCC TAT TTC CCA TGA-3’; sgRNA3 reverse 5’-GGT GAA GGA AGA CGT GCG GAA AAA AGC ACC GAC TCG G-3’. PCR products were cloned into pCR™-Blunt II-TOPO® (TOPO) using the Zero Blunt™ TOPO™ PCR Cloning Kit (Invitrogen). U6-sgRNA cassettes were excised with BbsI (NEB), gel purified using the Wizard® Genomic DNA Purification Kit (Promega) and used for Golden Gate assembly into shuttle plasmids pShuttle.Cre and pShuttle.CC9 containing sgRNA cloning site and expression cassettes for Cre or Cre-T2A-SpCas9. For the generation of shuttle plasmids, the Gateway™ pDONR^TM^221 plasmid (Invitrogen) was modified to include BsaI sites flanked by the attL sites. For pShuttle.Cre, the nls-Cre sequence was PCR amplified from pHR-CMV-nlsCre (Addgene #12265) using primers adding a BbsI site and a unique 4 bp sequence: nlsCre forward 5’-GGT GAA GGA AGA CGT CCG CGT TAC ATA ACT TAC GGT AAA TGG CCC GC-3’; nlsCre reverse 5’- GGT GAA GGA AGA CGA TTC CCT AAT CGC CAT CTT CCA GCA GGC GCA C-3’. The PCR product was cloned into pCR™-Blunt II-TOPO® using the Zero Blunt™ TOPO™ PCR Cloning Kit (Invitrogen). The previously modified Gateway™ pDONR^TM^221 plasmid and the BbsI-digested pCR™-Blunt II-TOPO®-nlsCre plasmid were used for Golden Gate Cloning together with a pre-annealed oligonucleotide containing two BsaI sites for later sgRNA insertion: forward 5’- GGC TAG AGA CCT AGA GCG ATC GCT CGC GGT CTC A-3’, reverse 5’- GCG GTG AGA CCG CGA GGC TAG CCT CTA GGT CTC T-3’. For the pShuttle.CC9 plasmid, nlsCre was PCR amplified using the nlsCre forward primer and a reverse primer containing part of a T2A sequence: nlsCre reverse-T2A: 5’- GGT GAA GGA AGA CGT TGT TAG CAG ACT TCC TCT GCC CTC TCC GCT TCC ATC GCC ATC TTC CAG CAG-3’. SpCas9 was amplified from pX330-U6-Chimeric_BB-CBh-hSpCas9 (Addgene #42230) using the primers: Cas9 forward-T2A 5’- GGT GAA GGA AGA CGA AAC ATG CGG TGA CGT CGA GGA GAA TCC TGG ACC TAT GGA CTA TAA GGA CCA CGA-3’, Cas9 reverse 5’- GGT GAA GGA AGA CGA TTC CCC AGC ATG CCT GCT ATT CTC TTC C-3’. Cre-T2A and T2A-Cas9 amplicons were cloned into pCR™-Blunt II-TOPO®, released by BbsI digest and used for Golden Gate Cloning as described for the pShuttle.Cre vector. Shuttle vectors containing sgRNAs were recombined with pAd/PL-Dest vector (Invitrogen) using the Gateway™ LR Clonase™ II Enzyme Mix (Invitrogen).

For generation of infectious adenoviruses (AVs), 10 µg pAd/PL-Dest vector, carrying the desired expression cassettes (sgRNAs, Cre, SpCas9), was linearized using PacI (NEB) to reveal ITR regions. The released adenoviral vector genome was purified using the Wizard® Genomic DNA Purification Kit (Promega) and transfected into 7 × 10^5^ Ad293 cells (Agilent) using Lipofectamine™ 2000 Transfection Reagent (Invitrogen). One day post transfection, medium was changed to DMEM supplemented with 2% FBS. Cells were harvested when showing the desired cytopathic effect and snap frozen in liquid nitrogen. AVs were released by 3 cycles of freeze–thaw and, following pelleting of debris (10 min, 3000 g, 4 °C), used to infect 8 × 15 cm dishes of 7 × 10^6^ Ad293 cells seeded one day prior to infection. After 3–4 days, high-titer AV was harvested by resuspending and pelleting cells. Cell pellets were resuspended in 5 ml PBS/10% Glycerol (Roth) and AV particles were released by 3 freeze–thaw cycles followed by centrifugation for removal of debris (10 min, 3000 g, 4 °C). For in vitro experiments, cells were incubated with AVs diluted in a low volume of DMEM supplemented with 2% FBS for 1 h before adding complete DMEM (10% FBS, 1% P/S) to full volume. For in vivo experiments, AVs were commercially (ViraQuest Inc.) amplified, purified and titrated for plaque-forming units (PFU).

### Luciferase assays

To monitor tumor development by GLuc secretion, 10–20 µl blood was obtained by puncturing the tail vein. Blood was directly mixed with 4 µl of 0.125 IU/ml heparin (Ratiopharm). Plasma was collected by centrifugation (15 min, 1200 g, 4 °C) and, optionally, stored in round-bottom 96-well plates sealed with clear foil at -20 °C. Luciferase activity measurements of plasma samples were performed as previously described for monitoring of transplanted tumors [27]. Briefly, plasma samples were diluted with phosphate-buffered saline (PBS) to match the dynamic range of the Orion II luminometer (Berthold). 5 µl of diluted plasma were transferred to white 96-well plates with V-bottom (Greiner) and measured by automated injection of 50 µl coelenterazine (PJK, Germany, stock diluted 1:200 in PBS). Coelenterazine was prepared as a 10 mM stock in acidified ethanol (10 ml EtOH + 200 µl 6 M HCl). To monitor the time course of tumor development and account for subtle differences in AV infection efficiency, all luminescence values from one mouse were normalized to its baseline luminescence, operationally defined as the mean luminescence during the first 90 days after AV infection. The mean ± 3SD of the baseline luminescence of all mice in the experiment was considered ‘background’. Normalized luminescence values exceeding this background level were considered significantly altered. For measuring luciferase activity in tissues, 10–20 mg tissue were lysed with 5 × Cell Culture Lysis Reagent (Promega) and a metal bead for 5 min at 50 Hz in TissueLyser LT (QIAGEN). Lysates were cleared from debris by centrifugation and measured as described for plasma samples.

### Western blot

For immunoblotting, cells were lysed in RIPA Lysis Buffer (50 mM Tris–HCl, 150 mM NaCl, 0.1% SDS, 1% Sodium Deoxycholate, 1% Triton X-100) supplemented with protease inhibitor (complete ULTRA tablets EASYpack, Roche). The following antibodies were used: anti-Cas9 (Diagenode #C15200216 1:500), anti-p53 (Bioss #bs8687R, 1:1000), anti-Rb1 (Cell Signaling #9313, 1:1000), anti-p130 (SantaCruz Biotech #sc-317, 1:200), anti-β-actin (AC-15, #ab6276, Abcam, 1:2500). For detection, secondary anti-rabbit or anti-mouse IgG-HRP (GE Healthcare, 1:5000) and SuperSignal ECL Kit (ThermoFisher) were used. Anti-β-actin was detected using an Alexa-488 coupled secondary antibody.

### Immunohistochemistry

For immunohistochemistry (IHC), tissue samples were cut as 3 µm thick sections from formalin-fixed paraffin embedded (FFPE) tissues. IHC staining was performed using a Bond Max automated staining system (Leica) using the antibodies: anti-Ascl1 (Abcam #211327, 1:400), anti-Chromogranin (Abcam #52983, 1:250), anti-Synaptophysin (Abcam #32127, 1:1000). GLuc and Cas9 staining was performed manually using the antibodies: anti-GLuc (Prolume Ltd, 1:1000), anti-Cas9 (Cell Signaling #19526, 1:400). Images were acquired using the Leica Aperio Versa slide-scanner and Leica Aperio eSlide Manager software v. 1.0.3.37. IHC images were analyzed quantitatively using the Aperio ImageScope software v. 12.3.2.8013. Tumors were marked and outlined by individuals blinded to the experimental setup and their area quantified in ImageScope. Tumor burden was calculated as percentage of tumor area to total lung area.

### CRISPR editing assays

*T7 Endonuclease I assay.* For analysis of the gene editing efficiency following infection with CRISPR AVs, sgRNA target sites were PCR amplified using genomic DNA of infected cells. PCR amplicons were purified using the PCR Purification Kit (QIAGEN) and analyzed by T7 Endonuclease I Assay as described [24]. Primers for *Trp53*: forward 5’-CGT CCA ATG GTG CTT GGA CA-3 ‘; reverse 5 ‘-GGG AAG AAA CAG GCT AAC CTA ACC-3’, *Rb1*: forward 5’- CTG CTG GGA TTA AAG GCA AG-3 ‘; reverse 5 ‘-CCT GCA CTC ACA CTC AGG AA-3’, *Rbl2*: forward 5’- GTA CTA CAC AAG GGT GTG GGC-3 ‘; reverse 5 ‘-CGA GGG GAG CCT GTT CTT ACA AAA-3’.

*CRISPR amplicon sequencing.* For sequencing analysis of gene editing events, sgRNA target sites were amplified from genomic DNA of cell culture, lung or tumor samples using the primers *Trp53*: forward 5’-CGA TGG TGA TGG TAA GCC CTC-3 ‘; reverse 5 ‘-TCT AGG CTG GAG TCA ACT GTC TC-3’, *Rb1*: forward 5’-AAG TAC ATT GCA GCA TCT TG-3 ‘; reverse 5 ‘-AGG TCA CTT ACG CAT GAA TA-3’, *Rbl2*: forward 5’- TCC AGA CCG GCA CCC TTT GTT C-3 ‘; reverse 5 ‘-TAC TGA CCT GCG CGT TTG CCT G-3’. For multiplex sequencing of multiple samples, amplicons were barcoded by adding the following overhangs to the gene-specific primers listed above: B1: forward 5’-TCA CTG GCA-3’; reverse 5’-TAG CTG CTG GCA-3’, B2: forward 5’-AGT GGT CGA-3’; reverse 5’-GTA CAT GGT CGA-3’, B3: forward 5’-CTA TAC TGT G-3’; reverse 5’-AGA GCA CTG TG-3’, B4: forward 5’-CGG ACA AAA G-3’; reverse 5’-TGT TCC AAA AG-3’, B5: forward 5’-GTC TAG CCA CC-3’; reverse 5’-ACT AGC CAC C-3’. PCR products were purified as described before and their concentration was determined using a NanoDrop™ 2000 spectrophotometer (Thermo Fisher). Barcoded PCRs for *Trp53*, *Rb1* and *Rbl2* were pooled and sequencing libraries were prepared using the NEBNext Ultra DNA Library Prep Kit (NEB) and sequenced on a MiSeq platform (Illumina) with the MiSeq Reagent Kit v3 (600-cycle) or MiSeq Reagent Kit v2 (500-cycles). Editing events were analyzed with CRISPResso2 [37].

*CRISPR Off-Target Analysis.* Possible off-target sites of the *Trp53*, *Rb1* and *Rbl2* sgRNAs were identified using the CRISPRoff Tool version 1.2 beta [38]. The list of predicted off-targets was filtered for intragenic location and presence of an “NGG” PAM and sorted by CRISPRoff score (Additional file 3). The Top10 hits for each of the *Trp53*, *Rb1* and *Rbl2* sgRNAs were PCR amplified from genomic tumor DNA (Additional file 4) and analyzed for mutations by Sanger sequencing.

### Software and statistical analysis

All statistical analyses were performed with GraphPad Prism 8 software. All graphs show mean values obtained with n biological replicates, and error bars in all figures represent standard deviation (SD), unless indicated otherwise. A *P*-value 0.05 was used as the threshold level for significance. For Kaplan–Meier survival curves, the log-rank test was applied. Two groups were tested for statistically significant differences by a two-sided unpaired t-test; multiple groups were tested by 1way ANOVA in conjunction with a post hoc multiple comparison test. Experimental schemes were generated with BioRender.com.

## Results

### Generation of conditional GLuc reporter mice

To enable in vivo labelling of cells with GLuc for blood-based monitoring of tumors, we have first generated a GLuc transgenic knock-in mouse *Gt(ROSA)26Sor*^*tm2(CAG−GLuc)Thst*^ (short: *Rosa26*^LSL−GLuc^ or LSL-GLuc) (Fig. 1a). GLuc expression is under control of the strong, ubiquitously active synthetic CAG promoter and rendered conditional to Cre recombinase activity by insertion of a *loxP*-flanked transcriptional stop cassette (LSL). To test the inducibility of GLuc in different organs, we excised the LSL-cassette from the germline by crossing LSL-GLuc and *Prm*-Cre mice [33] and compared GLuc activity in organ lysates from LSL-GLuc and GLuc littermates (Fig. 1b, c). GLuc activity in LSL-GLuc mice was not significantly different from background luminescence in non-GLuc-transgenic control mice, indicating that expression is not leaky (Fig. 1c). Removal of the LSL cassette led to strong induction of GLuc activity in all analyzed organs (Fig. 1b), exceeding the background level by, on average, more than 4 orders of magnitude (73,118-fold; *P* < 0.0001; Fig. 1c). For longitudinal monitoring of cells using GLuc blood levels, it is essential that GLuc is secreted at a constant rate resulting in stable blood activity levels over time. When monitoring the blood of animals over 4 months, luciferase blood activity in GLuc mice was more than 4 orders of magnitude higher than in non-Cre expressing LSL-GLuc mice (mean 13,595-fold; *P* < 0.0001), with little to no variation over time (Fig. 1d, e). Again, transgene expression was not leaky as blood levels in LSL-GLuc mice were not significantly different from controls (Fig. 1e). We conclude that the LSL-GLuc reporter mouse is suitable to monitor cellular processes by non-leaky expression and stable secretion of GLuc.

### In vivo labelling of tumors with GLuc

**Fig. 2:**
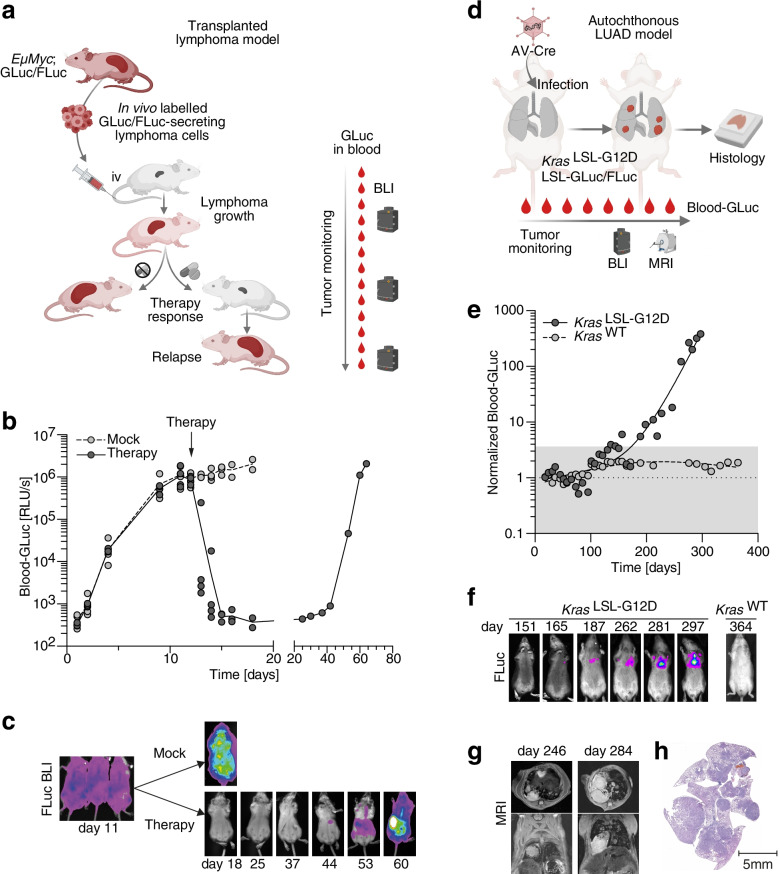
Monitoring classic tumor models with GLuc. a-c Monitoring of in vivo labelled transplanted lymphomas with GLuc. **a** Experimental scheme. Red color symbolizes GLuc activity. BLI, bioluminescence imaging. **b** Time course of GLuc activity in blood samples of mice transplanted with lymphoma cells from *EµMyc*;*Rosa26*^GLuc/FLuc^ mice. Data points represent individual mice. **c** BLI of representative mice at different time points after mock or cyclophosphamide treatment. **d-h** Monitoring of non-transplanted (autochthonous) lung adenocarcinoma with GLuc. **d** Experimental scheme. **e** Temporal development of blood GLuc activity in mice of indicated genotype. **f** Longitudinal BLI of mice with indicated genotype. **g** Sequential MRI of the *Kras*^LSL−G12D^ mouse. **h** H&E stain of the *Kras*^LSL−G12D^ mouse lung at time of sacrifice All error bars indicate SD, all data points represent biological replicates/individual mice. BLI, bioluminescence imaging; MRI, magnetic resonance imaging; LUAD, lung adenocarcinoma

In previous studies, tumor cells were labelled ex vivo with GLuc by, for example, lentiviral transduction to monitor their growth after transplantation into mice [20–22, 24, 27]. However, many primary tumor cells either fail to grow in culture or loose characteristic properties. For instance, Myc-induced Burkitt-like B cell lymphomas tend to develop chemotherapy resistance when cultured in vitro [39]. To test if luciferase-transgenic mice can be used for in vivo labelling and therapy monitoring of Myc-induced lymphomas, we generated *EµMyc* mice with GLuc and, for comparison, classical non-secreted firefly luciferase (FLuc) transgenes (Fig. 2a). Freshly explanted, in vivo GLuc/FLuc-labelled lymphoma cells were transplanted into recipients and disease development was monitored based on GLuc blood levels (Fig. 2b). GLuc activity progressively increased in the blood of all animals by more than 3 orders of magnitude by day 11, when lymphoma disease was independently confirmed by bioluminescence imaging (BLI) for FLuc activity (Fig. 2c). On day 12, half of the animals received a single dose of cyclophosphamide chemotherapy, causing a > 1000-fold decrease in GLuc blood levels over the next two days and absence of FLuc BLI signals on day 18. All untreated animals showed progressively increased GLuc blood levels and FLuc BLI signals before reaching the humane study endpoint with extensive lymphoma burden. One of the treated mice died from relapse after two months. This was preceded by a parallel increase in GLuc blood and FLuc BLI signals. These observations underline that in vivo labelling with both luciferases similarly enabled longitudinal monitoring of therapy responses. However, while the distress caused by anesthesia is limiting the BLI examination frequency, small-volume, 10–20 µl blood samples needed for GLuc activity measurements could be obtained much more frequently, yielding a high temporal resolution for capturing fast dynamic processes such as therapy responses.

We next explored using LSL-GLuc reporter mice for monitoring of non-transplanted tumors developing in an autochthonous model of *Kras*^G12D^ oncogene-driven lung adenocarcinoma. For this, we generated a mouse carrying a germline knock-in of the Cre-inducible *Kras*^LSL−G12D^ oncogene [30] in conjunction with the conditional LSL-GLuc and LSL-FLuc alleles (Fig. 2d). Upon intratracheal infection with Cre-expressing adenovirus (AV-Cre), the *Kras*^LSL−G12D^ mouse developed multiple lung adenomas and adenocarcinomas accompanied by a parallel increase in GLuc blood activity and thoracic FLuc bioluminescence 5–10 months after infection (Fig. 2e-h). Of note, a mouse carrying luciferase alleles but no oncogene did not show detectable increases in GLuc or FLuc activity following AV-Cre infection confirming a tumor-derived origin of the signals (Fig. 2e-f). Together these pilot experiments demonstrated the suitability of LSL-GLuc reporter mice for in vivo labelling of tumor cells and monitoring tumor growth.

### Flexible toolkit for rapid assembly of CRISPR-adenoviruses

The natural tropism for the respiratory epithelium makes adenoviral vectors (AVs) particularly efficient for lung-specific delivery of Cre and activation of Cre-inducible germline-encoded transgenes such as *Kras*^LSL−G12D^ [30, 40–42]. Moreover, the large transgene packaging capacity makes AVs also exceptionally well suited to deliver larger genetic cargo such as CRISPR nucleases for lung-specific induction of somatic cancer mutations [15, 43, 44]. However, the most commonly used adenovirus (serotype 5) consists of a large linear, 36-kb, double-stranded DNA molecule, which makes cloning adenoviral vectors more laborious than, for example, lentiviral or adeno-associated vectors. To more rapidly produce CRISPR-adenoviruses (CRISPR-AVs) delivering Cre-recombinase together with CRISPR nucleases, consisting of *Streptococcus pyogenes* Cas9 (SpCas9) and gene-specific sgRNAs, we have developed a cloning toolkit which uses Golden Gate cloning with type IIs restriction endonucleases [45] for the flexible assembly of multiple expression cassettes and Gateway recombineering [46] for the final integration of the complete multi-cistronic assembly into the adenoviral vector genome [47].

**Fig. 3:**
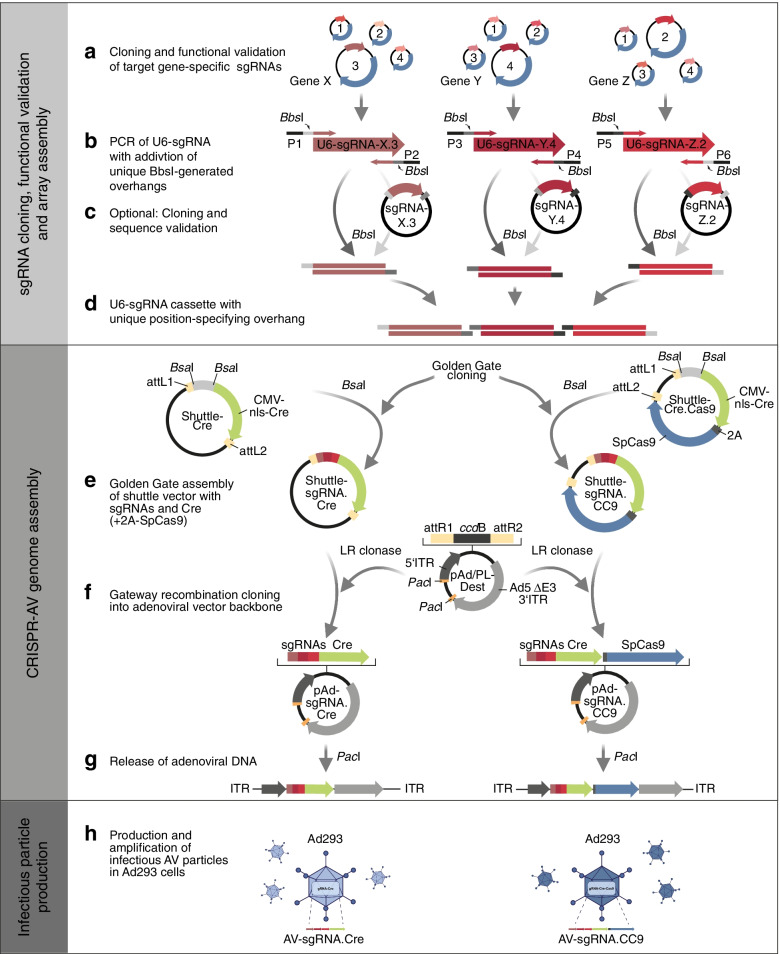
Toolkit for cloning CRISPR adenoviruses. **a** Multiple candidate sgRNAs targeting cancer genes of interest are cloned into plasmids co-expressing Cas9 and a puromycin resistance gene for functional validation in cell culture. **b** Selected validated sgRNA expression cassettes including U6 promoter and sgRNA scaffold are PCR amplified using primer pairs adding a BbsI recognition site and a 4-bp motif specifying the position in the final vector construct. **c** Optional cloning of PCR amplicons for sequence verification by Sanger sequencing. **d** Release of complementary overhangs by BbsI. **e** Golden Gate assembly of multiple BbsI-digested sgRNA-cassettes with BsaI-digested shuttle vectors containing expression cassettes for Cre + Cas9 or Cre only. **f** Gateway recombination cloning of modified sgRNA-containing shuttle vectors into the adenoviral vector backbone (pAd/PL-Dest destination vector). **g** Release of linear adenoviral DNA by PacI digest. **h** Transfection of Ad293 cells for production and amplification of infectious AV particles

In the first step, sgRNAs targeting the genes of interest are designed and cloned into the puromycin-selectable SpCas9-encoding pX459 plasmid [48] (Fig. 3a). For modelling small cell lung cancer (SCLC), we targeted *Trp53* and *Rb1*, the mouse homologues of the human genes *TP53* and *RB1*, which are mutated in > 90% of all SCLC patients [1]. For each target gene, multiple sgRNAs with low off-target scores (Additional file 3) were cloned into pX459, transfected into NIH3T3 mouse fibroblasts and evaluated for induction of insertion and deletion (indel) mutations at the genomic sgRNA target sites. Functionally validated U6 promoter-driven sgRNA expression cassettes were PCR-amplified from pX459 plasmids using primers containing binding sites for the type IIs restriction enzyme BbsI, which releases a unique 4-bp overhang for later assembly with other expression cassettes for sgRNAs, SpCas9 and Cre (Fig. 3b). As an optional step, the PCR amplicons were cloned into a compatible vector for sequence validation (Fig. 3c). Eventually, multiple sgRNA amplicons were digested with BbsI (Fig. 3d) and assembled into a BsaI-restriction site of a shuttle vector containing a CMV promoter-driven Cre-2A-SpCas9 expression cassette flanked by attL recombination sites for Gateway recombination cloning (Fig. 3e). The entire assembly comprising all sgRNAs, Cre and Cas9 was recombined into attR sites of a destination vector containing a first generation (E1/E3-deleted) adenoviral vector genome (Fig. 3f). For production of infectious viral particles, the recombinant adenovirus genome was released from the plasmid by PacI restriction digest (Fig. 3g) and transfected into Ad293 cells for multiple rounds of viral amplification and purification [40] (Fig. 3h). We have hereby developed a versatile cloning system for the rapid generation of adenoviral CRISPR vectors expressing different sgRNA combinations in conjunction with Cre and SpCas9.

### Monitoring CRISPR-induced lung tumorigenesis using GLuc blood-levels

**Fig. 4:**
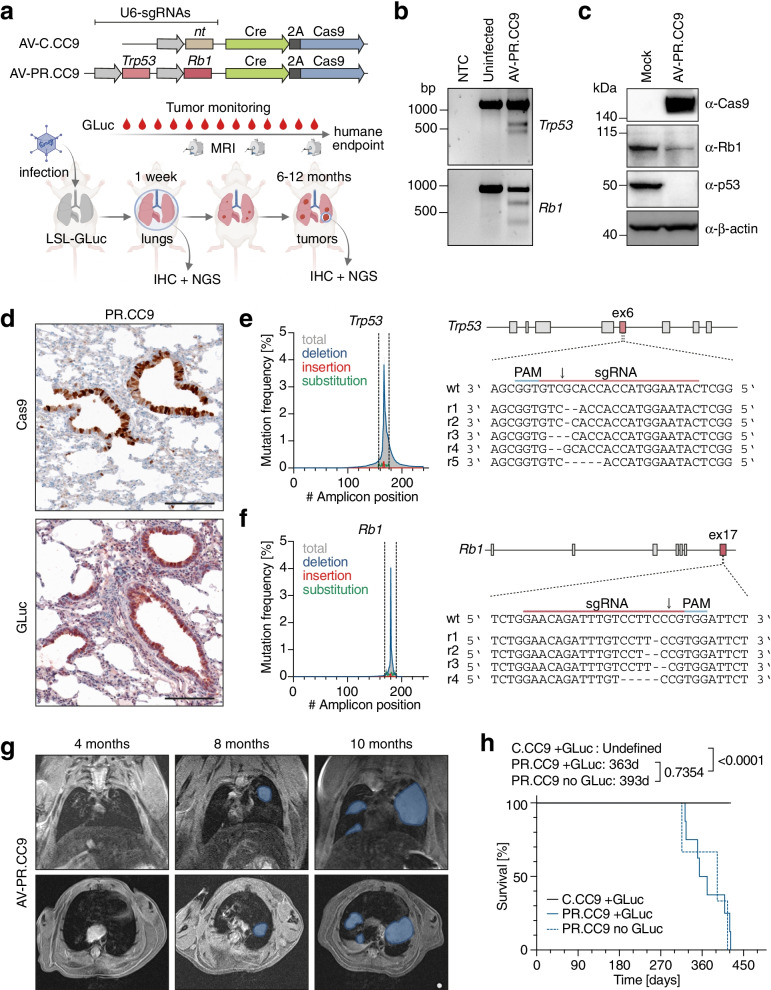
SCLC induction by adenoviral delivery of CRISPR nucleases. **a** Experimental scheme for SCLC induction and monitoring with adenoviral vectors (AV) expressing Cre and *Trp53*/*Rb1*-targeting Cas9 nucleases. **b** Validation of sgRNA function by T7 endonuclease I assay using genomic DNA from uninfected and AV-PR.CC9 infected NIH3T3 cells. NTC, no template control. **c** Western Blot of mouse embryonic fibroblasts (MEF) infected with AV-PR.CC9 showing Cas9 expression and downregulation of p53 and Rb1 protein levels. β-actin is shown as loading control. **d** Immunohistochemistry for GLuc and Cas9 expression in the lung of mice 1 week after intratracheal AV-PR.CC9 infection. **e,f** Mutation spectrum of **e** *Trp53* exon 6 and **f** *Rb1* exon 17 sgRNA target loci (flanked by dashed lines). Shown are sequencing reads of the most abundant indel mutations at the sgRNA target site. **g** Sequential MRI of a representative mouse showing tumor progression. Shown are frontal and transversal sections with tumors marked in blue. **h** Kaplan–Meier survival plot of WT and LSL-GLuc mice infected with indicated CRISPR-AVs (AV-C.CC9 + GLuc: *n* = 12; AV-PR.CC9 + GLuc: *n* = 8; AV-PR.CC9 no GLuc: *n* = 3). Shown are median survival and P-values from Log-rank (Mantel-Cox) test

We next investigated whether a *Trp53* (P) and *Rb1* (R) targeting CRISPR-AV (AV-PR.CC9) induces lung tumors that can be monitored using GLuc blood levels (Fig. 4a). Confirming the anticipated function, NIH3T3 fibroblasts infected with AV-PR.CC9 induced frameshift-causing *Trp53* and *Rb1* indel mutations as shown by T7 endonuclease assay (Fig. 4b) and resulted in markedly reduced protein expression of p53 and Rb (Fig. 4c). Next, LSL-GLuc mice were infected with AV-PR.CC9 by intratracheal injection. After 1 week, we detected widespread Cas9 and GLuc expression in lung sections by immunohistochemistry (Fig. 4d) and insertion/deletion mutations in *Trp53* and *Rb1* at the sgRNA target site by next generation sequencing (NGS) with a mutation frequency of 6.8% and 6.4%, respectively (Fig. 4e, f). More detailed evaluation of the indel spectrum revealed mostly 1 to 5-bp deletions that were larger and more heterogenous in *Trp53* than *Rb1* (Fig. 4e, f). In parallel, we infected LSL-GLuc mice with AV-C.CC9, which expresses a non-targeting control sgRNA. Tumor development was monitored for up to 15 months (Fig. 4g, h). While none of the AV-C.CC9 infected control mice developed tumors, AV-PR.CC9 mice monitored by magnetic resonance imaging (MRI) started showing tumor nodules 8 months post infection (Fig. 4g). Tumorigenesis proceeded with variable kinetics and reached the experimental endpoint after 9 to 15 months (Fig. 4h). The median survival of AV-PR.CC9-infected LSL-GLuc mice was not significantly different from infected wild-type mice (363 vs 393 days, *P* = 0.7354; Fig. 4h) and comparable to conditional *Trp53/Rb1* germline-mutant mouse models for SCLC [49, 50], indicating that neither GLuc expression nor the mechanism of mutagenesis alters the time course of SCLC development.

**Fig. 5:**
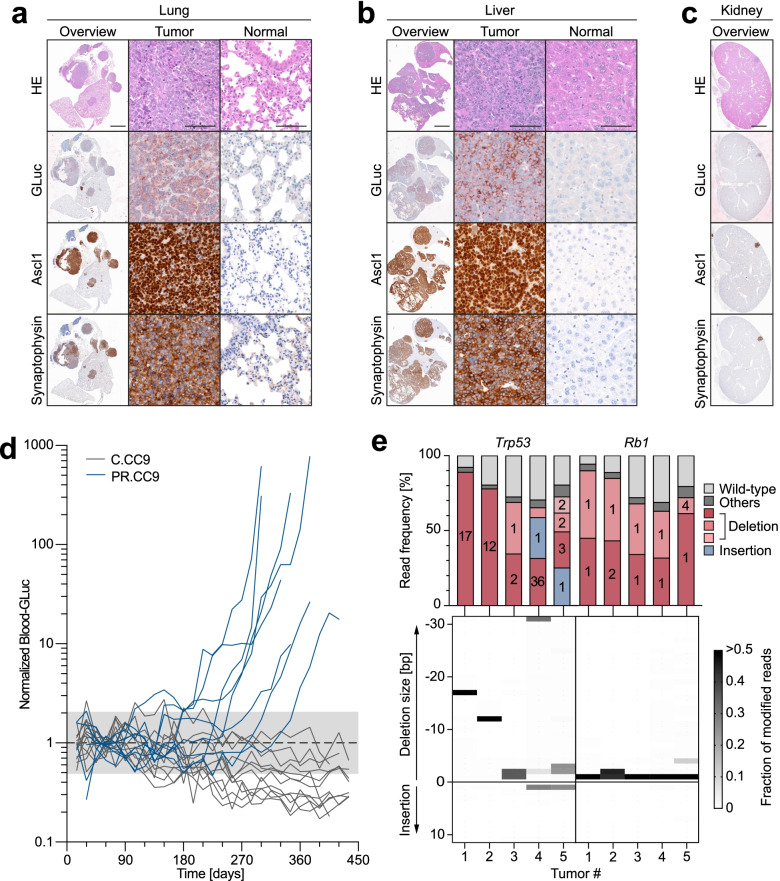
Molecular characterization and GLuc-based monitoring of CRISPR-induced SCLC. **a-c** Histological analysis of AV-PR.CC9 induced **a** primary lung tumors and metastases to the **b** liver and **c** kidney. Shown are representative H&E and immunohistochemical stains for GLuc and NE lineage markers (Ascl1, Synaptophysin). **d** Temporal development of blood GLuc activity in individual mice following infection with indicated AVs (AV-C.CC9 *n* = 12; AV-PR.CC9 *n* = 8). Shaded area represents the GLuc background activity. **e** *Trp53* and *Rb1* mutation spectra of single AV-PR.CC9 tumors from 5 different mice. Top graph, shown is the frequency of wild-type and mutant reads. For mutant reads, all mutations with a frequency of > 5% are color-coded as deletions or insertions and labelled with the number of deleted or inserted base pairs. Less frequent mutations are summarized as ‘others’. Bottom graph, shown is the size distribution of indel mutations for each tumor. The frequency is each indel mutations is encoded in grayscale

Histological examination of tumors showed the typical morphology of small cell lung cancer with multiple mitotic figures, dense sheets of tumor cells and fine granular chromatin (Fig. 5a). In line, the majority of tumors stained strongly positive for the neuroendocrine marker synaptophysin and the lineage-specifying transcription factor Ascl1 (Fig. 5a). At the time of sacrifice most animals displayed substantial metastasis, mostly to the liver and in some cases also to the kidney and ovary, showing expression of neuroendocrine markers similar to the primary lung tumors (Fig. 5b, c). Successful GLuc-labelling of tumor cells was confirmed by positive GLuc staining of lung tumor nodules and metastases (Fig. 5a-c). GLuc blood activity started to exceed background levels in individual mice as early as 5 months post infection and increased progressively by a mean 265-fold (range: 10 to 776-fold) until reaching the humane study endpoint (Fig. 5d). Immunohistochemistry confirmed that GLuc expression, similar to neuroendocrine tumor markers, was confined to tumor nodules and largely absent from the adjacent non-tumor tissues (Fig. 5a), strongly suggesting that GLuc activity in blood samples is predominantly derived from tumor cells rather than normal lung.

To confirm that tumors originate from cells with CRISPR-AV induced gene mutations, we sequenced the *Trp53* and *Rb1* genes of single tumors from 5 individual mice by NGS (Fig. 5e). In comparison to the low frequency of modified reads two weeks after adenoviral infection (Fig. 4e, f), tumors contained an average of 80% modified reads (*Trp53*: 0.79 ± 0.09; *Rb1*: 0.81 ± 0.11) (Fig. 5e). The mutant read frequency correlated significantly between the two genes (R^2^ = 0.88, *P* = 0.0188), suggesting that the tumors originate from double-mutant cells. The percentage of approximately 20% unmodified reads is in line with the expected percentage of non-tumor cells populating the tumor stroma. Most tumors showed one or two (equally frequent) dominant mutant sequences, underlining that tumors are clonal in origin. Tumors 4 and 5 showed additional less frequent mutant sequences, possibly derived from adjacent tumor nodules. Similar as previously observed in AV-PR.CC9-infected lungs 1 week post infection and consistent with erroneous DSB repair via NHEJ, all *Rb1* and *Trp53* tumor mutations were indel mutations. Compared to mostly frameshift-inducing small deletions of 1, 2 or 4 nucleotides in *Rb1*, *Trp53* indels were more variable in size and nature. In addition to frameshift-inducing deletions, we also observed several in-frame mutations of 3, 12 or 36 base pairs. As the targeted *Trp53* exon 6 encodes a part of the DNA binding domain of the p53 transcription factor, which is notoriously sensitive to even subtle mutations, these in-frame mutations are likely loss-of-function mutations deficient in tumor suppression. Together, the observed mutation spectrum confirms that the tumors monitored by GLuc blood levels are indeed resulting from the anticipated *Trp53* and *Rb1* mutations rather than potential off-target mutations.

### Comparative monitoring of genetically distinct tumor subtypes using GLuc blood-levels

**Fig. 6:**
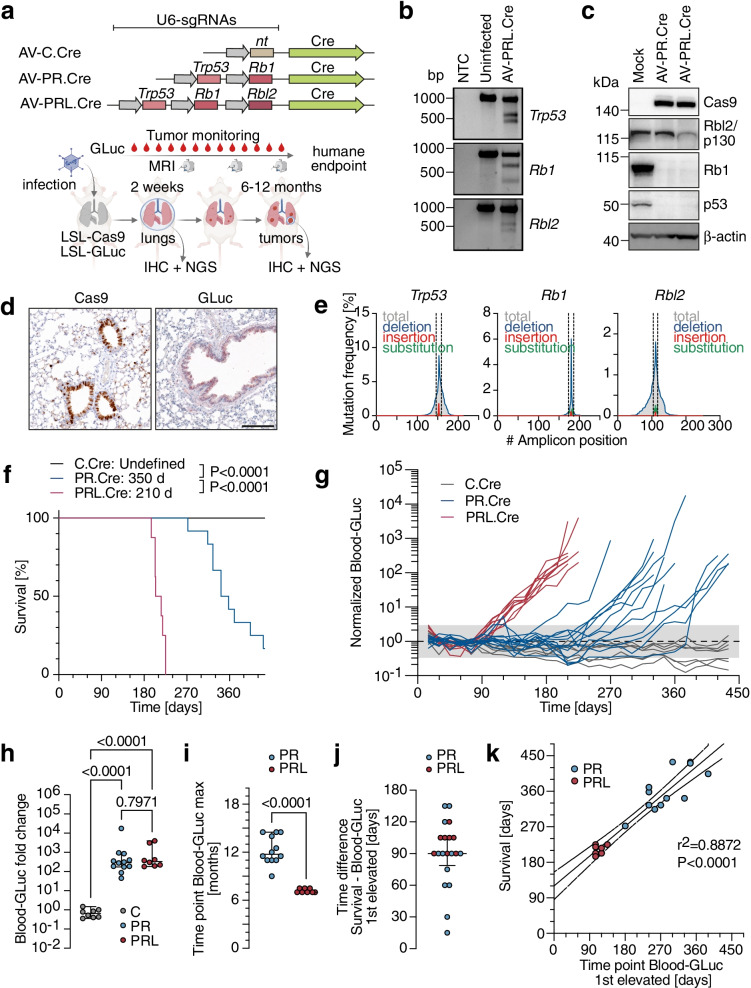
GLuc monitoring of SCLC induced by adenoviral sgRNA delivery to Cas9 mice. **a** Experimental scheme for SCLC induction and monitoring with adenoviral vectors (AV) expressing Cre and *Trp53*/*Rb1/Rbl2*-targeting sgRNAs. **b** Validation of sgRNA function by T7 endonuclease I Assay using genomic DNA from uninfected and AV-PRL.Cre infected NIH3T3-Cas9 cells. NTC, no template control. **c** Western Blot of LSL-Cas9 fibroblasts infected with AV-PR.Cre and AV-PRL.Cre showing downregulation of p53, Rb1 and Rbl2/p130 protein levels. β-actin is shown as loading control. **d** Immunohistochemistry for GLuc and Cas9 expression in the lung of mice 2 weeks post infection. Scale bar, 50 µm. **e** Mutation spectrum of *Trp53*, *Rb1* and *Rbl2* sgRNA target loci (flanked by dashed lines). **f** Kaplan–Meier survival plot of LSL-GLuc mice infected with indicated CRISPR-AVs (AV-C.Cre: *n* = 14; AV-PR.Cre: *n* = 12; AV-PRL.Cre: *n* = 8). Shown are median survival and P-values from Log-rank (Mantel-Cox) test. **g** Temporal development of blood GLuc activity in mice from **f** following infection with indicated AVs. Shaded area represents the GLuc background activity. **h** Total fold change in blood-GLuc activity over the course of tumor development. *P*-values from Tukey’s multiple comparisons test. **i** Time point when blood-GLuc activity reached its maximum. *P*-value from an unpaired, two-sided t-test. **j** Time difference between the time of sacrifice (survival) and the time point when blood-GLuc activity was first elevated, i.e. exceeded the background range. **k** Correlation between time of sacrifice (survival) and the time point when blood-GLuc activity was first elevated. Shown is the linear regression with 95% confidence interval All error bars indicate SD, all data points represent biological replicates/individual mice

We next explored whether GLuc-blood levels are suitable to monitor differences in tumorigenesis caused by distinct co-mutations. As a model we chose to monitor the impact of *Rbl2/p130* (L) mutations, which are recurrent co-mutations in SCLC patient tumors and accelerate SCLC tumorigenesis in mouse models [1, 15, 50]. Given the limited packaging capacity of 1^st^ generation AVs, insertion of a third sgRNA cassette into the SpCas9-Cre co-expressing AV vector backbone resulted in strongly reduced virus titers. To overcome the resulting delivery challenges, we crossed LSL-GLuc with LSL-Cas9 mice and used compound conditional transgenic LSL-Cas9/LSL-GLuc mice with Cre-inducible expression of GLuc and Cas9 [14]. This allowed us to omit Cas9 from the CRISPR-AV and instead introduce one or more additional sgRNA cassettes. Using this strategy, we generated CRISPR-AVs expressing Cre (without SpCas9) together with sgRNA combinations targeting *Trp53* and *Rb1* (AV-PR.Cre), *Trp53*, *Rb1* and *Rbl2* (AV-PRL.Cre) or a non-targeting control sgRNA (AV-C.Cre) (Fig. 6a). The AVs were validated in vitro by infection of Cas9-expressing NIH3T3 cells, confirming efficient induction of indel mutations at all three target loci (Fig. 6b). Infection of LSL-Cas9 fibroblasts showed successful Cre-mediated activation of Cas9 expression and depletion of the targeted gene products at the protein level (Fig. 6c).

Lung sections from AV-infected LSL-Cas9/LSL-GLuc mice showed efficient induction of Cas9 and GLuc expression (Fig. 6d) and accumulation of small *Trp53*, *Rb1* and *Rbl2* indel mutations (Fig. 6e). When monitoring infected mice for development of lung cancer symptoms, the PR.Cre group showed similar kinetics of tumorigenesis as our previous PR.CC9 group (median survival 350 days; Figs. 6f and 4h). In contrast, PRL.Cre infected mice showed a significantly reduced median survival of only 210 days (Fig. 6f). GLuc labelling did not seem to affect the outcome, as non-GLuc transgenic LSL-Cas9 mice infected with these AVs showed similar survival (Additional file 1). GLuc blood-levels increased in both PR.Cre and PRL.Cre groups by far more than 2 orders of magnitude until the time of sacrifice with no significant difference between the two groups (median fold change PR.Cre: 293-fold; PRL.Cre: 335-fold; *P* = 0.7971; Fig. 6g, h). In the C.Cre control group, GLuc blood-levels did not change significantly (median 0.6580-fold; *P* = 0.0846). Notably, although the fold change in GLuc blood-levels was not significantly different between PR.Cre and PRL.Cre mice, PRL.Cre mice reached the maximum GLuc blood-levels already after 7.2 ± 0.3 months, compared to 12.3 ± 1.8 months in PR.Cre mice (*P* < 0.0001, Fig. 6i), mirroring the differences in survival. Interestingly, GLuc blood activity exceeded the background level, operationally defined as the average activity in the first three months, by more than 3 standard deviations approximately 3 months (PR.Cre: 82 ± 38 days, PRL.Cre: 101 ± 11 days) before reaching its maximum level at the time of sacrifice (Fig. 6j). Importantly, this early detection time point correlated with the clinically defined experimental endpoint “survival” (r^2^ = 0.8872, *P* < 0.0001; Fig. 6k), validating GLuc blood levels as a suitable early detection marker for autochthonous lung tumors.

**Fig. 7:**
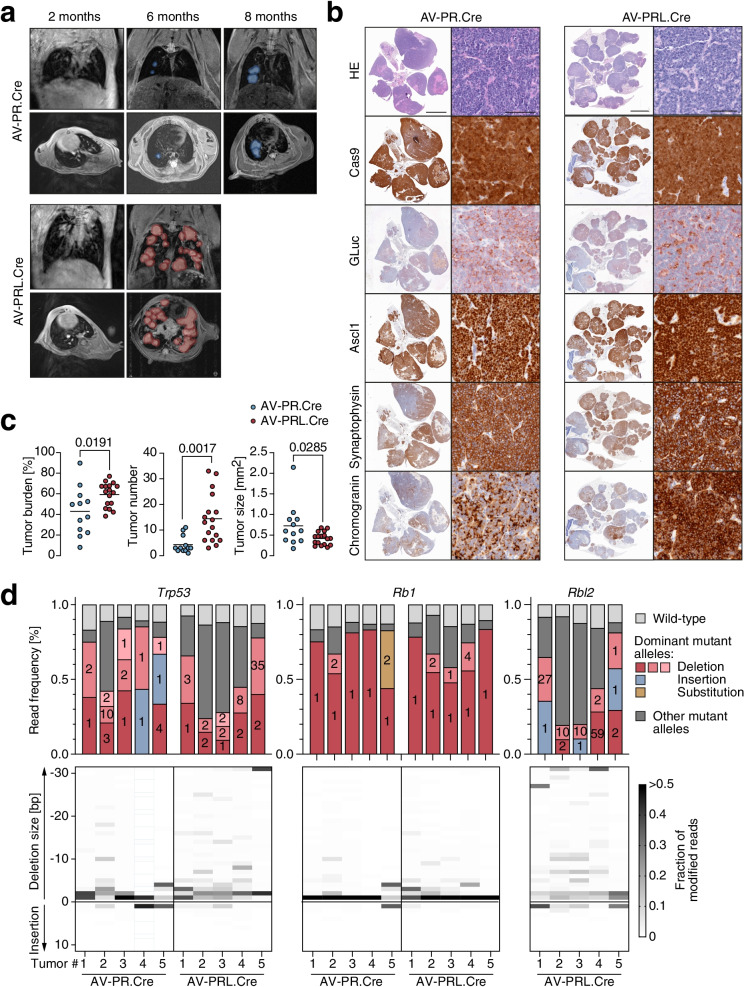
Rbl2 co-mutations accelerate SCLC tumorigenesis. **a** Sequential MRI of a representative AV-PR.Cre and AV-PRL.Cre infected mouse illustrating different kinetics of tumorigenesis. Shown are frontal and transversal sections with tumors marked in color. **b** Histological analysis of AV-PR.Cre and AV-PRL.Cre induced lung tumors. Shown are representative H&E and immunohistochemical stains for Cas9, GLuc and NE lineage markers (Ascl1, Synaptophysin, Chromogranin). **c** Quantitative analysis of SCLC tumor burden, tumor number and tumor size in AV-PR.Cre (*n* = 12) and AV-PRL.Cre (*n* = 17) infected mice. Shown is mean ± SD and P-values from unpaired, two-sided t-tests. **d** *Trp53*, *Rb1* and *Rbl2* mutation spectra of single tumors from 5 different mice of each group. Top graph, shown is the frequency of wild-type and mutant reads. For mutant reads, all mutations with a frequency of > 5% are color-coded as deletions or insertions and labelled with the number of deleted, inserted or substituted base pairs. Less frequent mutations are summarized as ‘others’. Bottom graph, shown is the size distribution of indel mutations for each tumor. The frequency of each indel mutations is encoded in grayscale

MRI of representative mice suggested that the *Rbl2* co-mutation increases both the number of developing tumors and their growth rate (Fig. 7a). This impression was confirmed by histological examination of lungs at the time of sacrifice (Fig. 7b, c). Although mice from the PRL.Cre group survived 5 months shorter, quantitative image analysis revealed a 1.4-fold higher tumor burden (*P* = 0.0191), which was mostly attributable to an increased number of tumors (3.0-fold, *P* = 0.0017) that were only slightly smaller in size compared to tumors from PR.Cre mice (0.61-fold, *P* = 0.0285; Fig. 7c).

Tumors from both groups expressed GLuc and the whole set of neuroendocrine markers, characterizing both groups as small cell lung cancer (Fig. 7b). Since Cas9 is constitutively expressed after Cre recombination, IHC confirmed this by positive Cas9 staining of tumor sections (Fig. 7b). Sequencing analysis of tumor nodules of 5 mice from each group showed *Trp53* and *Rb1* mutant allele frequencies of > 80% in all samples (Fig. 7d). Importantly, all PRL tumors also showed > 80% of mutant *Rbl2* reads identifying triple mutant cells as the cell of origin. Moreover, sequencing of predicted sgRNA off-target sites detected only in one of the tumors a single intronic base substitution (Additional file 3), excluding off-target editing as a cause of enhanced tumorigenesis in the PRL group. As seen in our previous PR.CC9 group (Fig. 5e), *Rb1* mutations were mostly small 1 bp deletions (Fig. 7d). In contrast, *Trp53* and *Rbl2* indel mutations were more diverse in size, allowing deeper insight into the clonal architecture of each analyzed tumor. While the majority of PR tumors showed 2 mutant *Trp53* alleles, PRL tumors more often contained a higher number of different *Trp53* and *Rbl2* mutations. When sequencing cell lines established from explanted SCLC tumors, the frequency of wild-type reads strongly decreased (Additional file 2). However, PRL cell lines still showed a higher number of different indel mutations than PR cell lines (Additional file 2). This strongly suggests that PR tumors are mostly monoclonal in origin, whereas PRL tumors more often consist of more than one clone. The number of tumors calculated by histological image analysis (Fig. 7c) is therefore likely an underestimation of the true number of tumor clones growing in the lungs of the PRL.Cre mouse group.

We conclude that blood-based GLuc monitoring provides an inexpensive and simple to implement technique for accurately assessing the impact of genetic factors such as co-mutations on tumor development and growth.

## Discussion

Over the last decade, mouse models for cancer have improved significantly, mimicking increasingly well the spontaneous processes of tumor development, initiated by the genetic transformation of somatic cells at their natural site of origin [8]. In particular, the application of somatic mutagenesis with CRISPR nucleases has been a gamechanger that makes modelling of human cancer mutations in the mouse easier than ever [9, 10]. As more and more personalized treatment approaches with molecular drugs are developed, mouse models that accurately recapitulate the genetics of the human disease become essential preclinical tools. Nevertheless, the majority of preclinical research, especially drug testing, is still performed using subcutaneously transplanted xenograft tumors, as it was common practice already decades ago. A major reason is that tracking subcutaneous tumor growth is inexpensive and quick, whereas monitoring the development and orthotopic tumor growth in inner organs requires sophisticated small animal imaging technology and a highly trained staff [51]. Even though imaging is largely considered a non-invasive refinement method according to the 3R principles, it relies on anesthesia to restrain the animals and their gross motion and often also requires injection of contrast agents, tracers or luminescence substrates to visualize the tumor properly. This increases the image acquisition time by the anesthetic induction and recovery time and strongly reduces the possible throughput in cohort studies [52, 53]. Moreover, repeated anesthesia required for longitudinal studies, the exposure to ionizing radiation and the use of contrast agents also have consequences on the physiology of the animal and impose still poorly understood physical and mental distress on the animals [53, 54]. To make better use of the more human-like cancer disease models for preclinical studies, monitoring techniques are needed that provide a higher throughput at a lower cost—ideally while simultaneously minimizing distress to the animals.

An alternative to imaging is monitoring of tumor growth using biomarkers secreted by tumor cells into the blood. The monitoring of tumor-related marker proteins has been routine practice in cancer screening and clinical monitoring of cancer patients for relapse since decades [55] and has attracted even more attention in the recent years as liquid biopsy-based, ctDNA analyses have emerged as an effective strategy for non-invasive genetic cancer assessment in many stages of patients’ monitoring [56]. However, not all tumors secrete specific marker proteins and the amount of blood needed for ctDNA analysis is restricting its use in small animal models. Different from the clinical tumor markers and ctDNA, secreted luciferases have the advantage of an excellent signal-to-noise ratio, a high dynamic range over several orders of magnitude, and—being actively secreted in an energy-consuming process—a direct relationship of signal to cell viability [20, 57]. As such, secreted *Gaussia* and *Cypridina* luciferases enable a blood-based tumor monitoring with high sensitivity and specificity using small-volume (10-20 µl) blood samples [20, 21, 27]. However, this requires the tumor cells to be labelled with the luciferases which is commonly achieved by transfection or retroviral transduction prior to their implantation into mice. While this works excellent for various transplanted mouse tumor models [22, 25, 26], the need for ex vivo labelling has prevented its use in autochthonous tumor models. The conditional GLuc-transgenic reporter mouse, developed in our study, overcomes this limitation and allows in situ labelling of cells by temporospatially controlled expression of Cre recombinase. Coupling Cre-mediated GLuc induction to tumor induction is achieved either by simultaneous Cre-mediated recombination of germline-encoded mutations, as demonstrated in the *Kras*^G12D^-driven lung adenocarcinoma model (Fig. 2d-h), or by co-delivery of Cre with components of cancer-inducing CRISPR nucleases, as demonstrated in the SCLC models (Figs. 4, 5, 6 and 7). Importantly, in vivo GLuc labelling does not alter the disease time course as wild-type and LSL-GLuc mice with CRISPR-induced SCLC have indistinguishable survival and tumor phenotype (Fig. 4h). Notably, tumor cells which cannot be propagated or loose characteristic properties in vitro, can be labelled in vivo with the GLuc transgene and then directly allografted into cohorts of experimental animals for preclinical drug studies (Fig. 2a-c).

Of note, not all AV-infected cells that recombine the GLuc transgene develop into a tumor and will generate a background of non-transformed GLuc expressing cells. In our experiments, AV infection increased the background GLuc luminescence of wild-type or non-infected mice (Fig. 1d) by approximately fivefold and this level was maintained for at least 3 months. We exploited the increase in background luminescence as an indicator of successful AV infection and compensated for it in the analysis by normalizing all GLuc measurements to the post-infection background activity level. In the later course of tumorigenesis, blood-GLuc activity increased further by more than 4 orders of magnitude until the humane endpoint was reached, highlighting a high dynamic range that is not compromised by the initial infection-related increase in luminescence background. In contrast, GLuc background activity decreased over time in non-tumor control mice (Figs. 5d, 6g), suggesting that GLuc-expressing epithelial cells are continuously replaced by non-recombined, GLuc-negative progenitors or stem cells.

Despite providing a highly quantitative measure of the viable tumor load in each animal, secreted luciferases are not ideal for localizing the tumors by imaging. In principle, tumors can be imaged using GLuc [20], but as tumors grow larger and blood-GLuc levels increase, systemically applied luciferase substrate is often completely metabolized in the blood before reaching the tumor so that the tumor signal is effectively disguised [27]. Nevertheless, GLuc-based monitoring of tumor load can be combined with bioluminescence imaging (BLI) using mice double transgenic for a secreted and a non-secreted luciferase, provided that both luciferase signals can be discriminated. As GLuc metabolizes coelenterazine, we used luciferin-consuming firefly luciferase (FLuc) as a non-secreted partner-luciferase for imaging (Fig. 2). While BLI and blood-GLuc measurements provided congruent results, BLI was temporally restricted to one examination per week because of animal welfare regulations. In contrast, small-volume blood samples of up to 1% of the animal’s total blood volume, i.e. up to 20 µl in mice, are allowed to be drawn daily for a period of two weeks [58], giving a much higher temporal resolution of tumor load measurement and facilitating studies into the dynamics of fast processes such as tumor therapy (Fig. 2b). Alternatively, tumors can also be imaged by other technologies such as MRI. While we could only analyze some exemplary animals with multiple techniques in parallel, these animals showed similar results (Fig. 2e-g). A perfect correlation, however, would not even be expected considering that morphological imaging by MRI or CT insufficiently discriminates viable and necrotic tumor volumes and that BLI signals are strongly influenced by light absorption dependent on the emitted wavelength, tissue depth and type and fur color [59]. On the other side, blood-GLuc activity might be affected by differences in tumor vascularization, an issue that has so far not been explored, but should be considered when testing, for example, anti-angiogenic drugs.

Another important consideration for preclinical drug studies is that tumors need to be detectable early enough before the humane endpoint is reached to provide a time window sufficiently large to evaluate therapy responses. As in particular immunotherapies often show first therapeutic effects only several weeks after the first dose, this issue becomes increasingly important as research into immunotherapies is exploding. Notably, blood-GLuc levels were first elevated in our SCLC models on average 3 months before the animals reached the humane endpoint (Fig. 6j) and this early detection timepoint correlated significantly with the survival time of the animal (Fig. 6k). Especially in autochthonous tumor models, where the time course of tumor development varies strongly between different animals, blood-GLuc levels appear optimal to repeatedly screen larger animal cohorts for disease onset with minimal cost and effort. Once tumor growth is evident in blood samples, blood-GLuc monitoring could be complemented specifically by more sophisticated imaging techniques if, for example, exact tumor localization is required. Similarly, blood-GLuc monitoring can be implemented to screen therapy cohorts of treated mice long-term for evidence of relapse, as demonstrated in Fig. 2b, again followed by complementary imaging techniques to localize the site of relapse (Fig. 2c).

Tumor induction by CRISPR-induced somatic cancer gene mutations is a rising technology, that not only suffers from easily accessible monitoring strategies but also from nuclease delivery issues [9, 10]. Adenoviral vectors (AVs) with their natural tropism for the respiratory epithelium are optimally suited for highly efficient gene transfer to the lung. Moreover, mouse cells are naturally non-permissive to human adenovirus replication providing an additional level of safety in mouse experiments. In addition, owing to the large size of their genome, already first-generation AVs have a sufficiently large packaging capacity to deliver SpCas9 nucleases together with Cre for lung tumor induction (Figs. 4 and 5). However, the large size of the genome also makes AVs more difficult to engineer than smaller lentiviral or adeno-associated vectors. We have therefore provided a flexible cloning toolkit to rapidly assemble AVs for expression of Cre, SpCas9 and variable combinations of sgRNAs. Of note, while AV genomes could be assembled that express three sgRNA cassettes together with Cre and SpCas9, these were not efficiently packaged into viral particles. For expression of three (or more) sgRNAs we therefore recommend using LSL-Cas9 mice in conjunction with an AV that expresses sgRNAs and Cre only. Although Cas9 will be expressed constitutively in this case (Fig. 7b), adenoviral transgene (sgRNA) expression is only transient, thus intrinsically preventing long-term nuclease activity. In fact, we did not observe differences in the kinetics and efficiency of tumor induction comparing SCLC induction with either AV-PR.CC9 (Figs. 4 and 5) or AV-PR.Cre (Figs. 6 and 7). Alternatively, Cre could be co-delivered with the nuclease and three or more sgRNAs by a single AV by switching to smaller orthogonal CRISPR nucleases (such as SaCas9, Cpf1 or CasMINI), to smaller crRNA arrays cleaved in vivo by Cpf1 or endogenous RNases or to helper-dependent ‘gutless’ AVs with almost unlimited packaging capacity [12, 13, 60–62].

In all SCLC tumors that were analyzed by deep sequencing, the total mutant read frequency exceeded 70%, consistent with a small subpopulation of genetically wild-type stromal and immune cells (Figs. 5e and 7d). The vast majority of mutations were deletions, followed by a smaller number of insertions and substitutions. In all cases, these occurred at the sgRNA target site and were predicted to disrupt protein function either by inducing a frameshift or by mutating or deleting functionally critical residues. Each PR tumor contained one to two dominant mutant alleles in the two target genes, indicating a clonal origin of the tumor nodule. In the case of PRL tumors, most tumors contained more than two dominant alleles in each of the three target genes, suggesting that the analyzed tumor nodules originated from more than a single cell. This is likely attributable to the previously reported high efficiency of SCLC induction by additional inactivation of *Rbl2* [15, 50], a gene known to be essential for maintaining cell cycle quiescence [63]. Together the deep sequencing analysis confirmed that the SCLC development measured by increasing blood-GLuc levels originated from tumors containing the desired gene mutations and accurately captures differences in tumorigenesis resulting from different sets of CRISPR-induced driver mutations.

## Conclusion

Our study describes a new GLuc reporter mouse for monitoring autochthonous tumors using luciferase measurements in blood samples. In addition, we provide a flexible toolkit for generating adenoviral vectors that, when used in conjunction with the conditional GLuc reporter mouse, simultaneously induce somatic mutations in cancer driver genes and label the resulting tumors with GLuc for blood-based monitoring. The combination of reporter mouse and adenoviral vector system will facilitate the use of autochthonous mouse tumor models in preclinical research by making tumor screening and monitoring of genetically-engineered orthotopic tumors considerably less time-consuming, less expensive and less burdening for the animals.

## Supplementary Information

**Additional file 1: **Survival of non-GLuc transgenic SCLC mice. LSL-Cas9 mice (without GLuc transgene) were intratracheally infected with either AV-C.Cre, AV-PR.Cre or AV-PRL.Cre as depicted in Fig. 6a. Shown is the Kaplan-Meier survival plot with group size, median survival and P-values from Log-rank (Mantel-Cox) test

**Additional file 2: **Mutation spectra of SCLC cell lines. Cell lines were established from AV-PR.Cre (*n*=3) and AV-PRL.Cre (*n*=5) SCLC tumors and analyzed by deep sequencing of the sgRNA target regions in* Trp53*, *Rb1* and *Rbl2*. Depicted is the frequency of wild-type and mutant reads. For mutant reads, the Top5 mutations are color-coded as deletions or insertions and labelled with the number of deleted, inserted or substituted base pairs. Less frequent mutations are summarized as ‘others’.

**Additional file 3: **sgRNA off-target analysis. *Trp53*, *Rb1* and *Rbl2* sgRNAs were analyzed for potential off-targets using the CRISPRoff Tool version 1.2. Shown are the Top10 hits with an intragenic location and “NGG” PAM. The Top10 hits for each of the *Trp53*, *Rb1* and *Rbl2* sgRNAs were PCR amplified using genomic tumor DNA from 3 AV-PR.Cre and 3 AV-PRL.Cre infected mice. PCR amplicons were analyzed for mutations by Sanger sequencing. Results are shown color-coded. Identified on-target and off-target mutations are shown in detail. Off-target mutations were all heterozygous missense mutations.

**Additional file 4: **Primers used for sgRNA off-target analysis.

## Data Availability

All of the materials, reagents and data generated during the current study are available from the corresponding authors upon request.

## Abbreviations

AV: Adenoviral vector
AAV: Adeno-associated virus
GLuc: Gaussia princeps luciferase
FLuc: Firefly luciferase
GEMM: Genetically engineered mouse model
LSL: LoxP-Stop-loxP
SCLC: Small cell lung cancer
NSCLC: Non-small cell lung cancer
CRISPR: Clustered Regularly Interspaced Short Palindromic Repeats
Cas9: CRISPR-associated 9 protein
sgRNA: Single guide RNA
TME: Tumor microenvironment
BLI: Bioluminescence imaging
MRI: Magnetic resonance imaging
CT: Computed tomography
NGS: Next generation sequencing
IHC: Immunohistochemistry
