# Supplementary figures and images for: Monitoring autochthonous lung tumors induced by somatic CRISPR gene editing in mice using a secreted luciferase
Source: Mol Cancer. 2022 Oct 3;21:191. doi: 10.1186/s12943-022-01661-2 (PMC9531476; doi:10.1186/s12943-022-01661-2)

Supplemental Figure 1

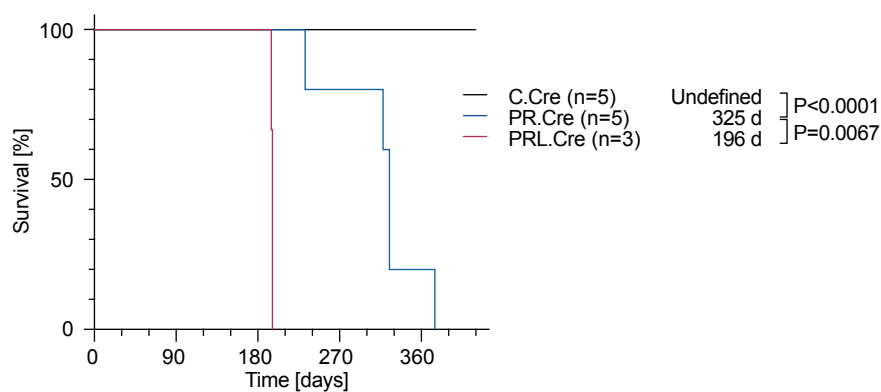

Supplement: Supplementary file 1 — Additional file 1: Survival of non-GLuc transgenic SCLC mice. LSL-Cas9 mice (without GLuc transgene) were intratracheally infected with either AV-C.Cre, AV-PR.Cre or AV-PRL.Cre as depicted in Fig. 6a. Shown is the Kaplan-Meier survival plot with group size, median survival and P-values from Log-rank (Mantel-Cox) test [file 12943_2022_1661_MOESM1_ESM.pdf]

Supplemental Figure 2

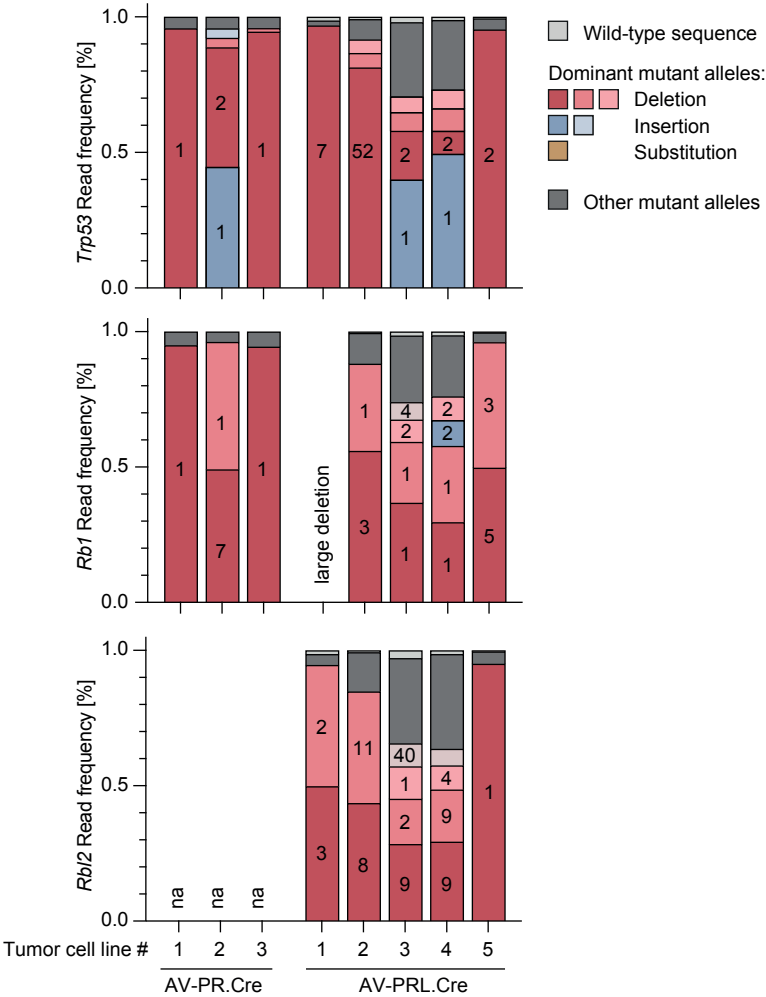

Supplement: Supplementary file 2 — Additional file 2: Mutation spectra of SCLC cell lines. Cell lines were established from AV-PR.Cre (n=3) and AV-PRL.Cre (n=5) SCLC tumors and analyzed by deep sequencing of the sgRNA target regions in Trp53, Rb1 and Rbl2. Depicted is the frequency of wild-type and mutant reads. For mutant reads, the Top5 mutations are color-coded as deletions or insertions and labelled with the number of deleted, inserted or substituted base pairs. Less frequent mutations are summarized as ‘others’. [file 12943_2022_1661_MOESM2_ESM.pdf]
